# Serum fatty acid binding protein 4 is positively associated with early stroke recurrence in nondiabetic ischemic stroke

**DOI:** 10.18632/aging.101886

**Published:** 2019-04-09

**Authors:** Bo Li, Jun Wu, Pengjun Jiang, Maogui Li, Qingyuan Liu, Yong Cao, Shuo Wang

**Affiliations:** 1 Department of Neurosurgery, Beijing Tiantan Hospital, Capital Medical University, Beijing, China; 2 China National Clinical Research Center for Neurological Diseases, Beijing, China; 3 Center of Stroke, Beijing Institute for Brain Disorders, Beijing, China; 4 Beijing Key Laboratory of Translational Medicine for Cerebrovascular Diseases, Beijing, China

**Keywords:** adipocyte fatty acid–binding protein, stroke recurrence, ischemic stroke

## Abstract

Adipocyte fatty acid–binding protein (FABP4) played critical roles in metabolic syndrome, inflammatory responses and cardiovascular diseases. It aimed to investigate the associations of serum FABP4 levels with early stroke recurrence. This study included the 206 acute ischemic stroke patients hospitalized in our institution. Stroke recurrence events were assessed at the 3-month follow-up. The median of FABP level was 22.6 (IQR, 17.9-31.6) ng/mL in patients with stroke recurrence (N=36), which was higher than in patients without stroke recurrence [16.9 (IQR, 11.8-21.4) ng/mL] (P<0.001). As a continuous variable, the unadjusted and adjusted risk of stroke recurrence would be increased by 12% (OR=1.12 [95% CI 1.06–1.17], P<0.001) and 8% (1.08 [1.02–1.14], P=0.006) for every 1 ng/ml increment of FABP4. The Area under the curve (AUC) of serum FABP4 and NIH Stroke Scale (NIHSS) score for predicting stroke recurrence was 0.73 (95% CI: 0.64–0.82) and 0.72 (95% CI: 0.64–0.81), presenting no discriminating capacity (P=0.45). In the combining model, the AUC of NIHSS score was further improved to 0.77 by FABP4 (0.77; 95% CI: 0.69–0.85), which was significant (P=0.01). The risk of stroke recurrence can be predicted by elevated FABP4 levels in serum of nondiabetic patients with first-ever ischemic stroke.

## Introduction

Stroke has been the cause of approximately 5% of all disability-adjusted life-years, as well as 10% of all mortality globally [1]. In China, stroke lead to nearly 1.6 million deaths each year, and there are also 7.5 million people survived from stroke [2]. Furthermore, 15%-30% of those survivors would be suffered from life-long disability [2]. There was study reported that in 2016, global lifetime risk of stroke could be 25% since the age of 25 years. Thereinto, the estimated risk was highest for China, which was 39.3% (95% uncertainty interval, 37.5 to 41.1) [3].

Adipocyte fatty acid–binding protein (FABP4/aP2) has been reported to function in metabolite and inflammation [4]. Previous studies performed on FABP4-deficient mice indicated that this lipid chaperone made important effects on metabolic syndrome [5], insulin resistance [6] and atherogenesis [7]. Another study suggested that aP2-targeted small-molecule inhibitors may be a new therapy for the prevention and treatment of metabolic diseases (e.g. type 2 diabetes or atherosclerosis) [8]. Furthermore, clinical research also suggested that FABP4 played critical roles in macrophage cholesterol trafficking and inflammation, and may promote the obesity development [9], insulin resistance [10], metabolic syndrome [11], diabetes [12], gestational diabetes mellitus [13], hypertension [14] and atherosclerosis [5,15–17].

Interestingly, one study reported that serum FABP4 levels in elderly population were related to an elevated risk of developing central arterial stiffness [18]. A community-based cohort study reported that the cardiovascular diseases could be predicted with the circulating FABP4 level [19]. Furthermore, a case-control study [20] indicated that ischemic stroke was significantly correlated with serum FABP4, which might be applied as prognostic indicator for early mortality. In addition, FABP4 was also related to various poor outcomes in patients with acute ischemic stroke [21]. Above results indicated the potential role of FABP4 in stroke. Thus, we hypothesized that FABP4 level in serum was related to the prevalence of stroke recurrence in nondiabetic ischemic stroke. The serum FABP4 levels were in 206 Han Chinese nondiabetic patients with ischemic stroke, and the associations between serum FABP4 levels and stroke recurrence in 3-month follow-up were explored.

## RESULTS

### Descriptive characteristics of stroke patients

324 patients with ischemic stroke were included. 255 patients were recorded after excluding 45 with diabetes mellitus, 8 with tumors, 6 inflammatory disease and 10 with abnormal renal and liver function. There were 9 lost to follow-up and 5 withdrew. Finally, 241 patients completed follow-up and 206 patients were included (35 patients died during follow-up). The FABP levels of those patients were collected and analyzed. The median value of FABP levels was calculated as 17.7 (IQR, 12.7–22.8) ng/ml and the median value of age was 59 (IQR, 48-73) years. The median NIHSS score was 7 (IQR, 4-12) points during their hospitalization. The baseline information of all patients was summarized (Table 1).

**Table 1:** Baseline characteristics of 206 patients with stroke.

	N=206
Age (years), medians (IQRs)	59(48-73)
Sex-male, n (%)	112(54.4)
BMI (kg/m2), medians (IQRs)	26.7(24.8-28.2)
Time from onset to blood collection(hours), medians (IQRs)	22.5(13.0-28.5)
Vascular risk factors, n (%)	
Hypertension	141(68.4)
Hypercholesterolemia	55(26.7)
Atrial fibrillation	46(22.3)
Coronary heart disease	22(11.7)
Previous TIA	25(12.1)
PVD	11(5.3)
Acute treatment, no. (%)	
TPA-T	52(25.2)
Mechanical thrombectomy	9(4.4)
Mechanical thrombectomy and/or TPA-T	57(27.7)
NIHSS at admission, medians (IQR)	7(4-12)
Lesion volumes (ml), median (IQR)	21.8(8.6-35.9)
Stroke etiology no. (%)	
Small-vessel occlusive	42(20.4)
Large-vessel occlusive	40(19.4)
Cardioembolic	77(37.4)
Other	20(9.7)
Unknown	27(13.1)
Laboratory findings, medians (IQR)	
Hs-CRP, mg/dl	0.74(0.27-1.87)
HCY, umol/l	16.6(12.4-20.5)
FABP4, ng/ml	17.7(12.7-22.8)

IQR, interquartile range; NIHSS, National Institutes of Health Stroke Scale; TPA-T: Tissue plasminogen activator-treated; Hs-CRP, high C-reactive protein; HCY, homocysteine; TIA, Transient ischemic attack; PVD, peripheral vascular disease; FABP4; Fatty Acid Binding Protein 4.

### Main results

**Figure 1:**
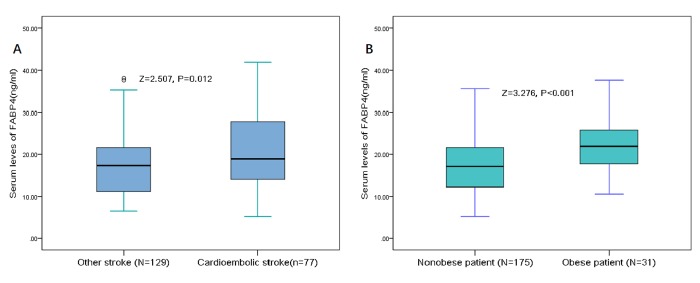
**Distribution of serum levels of FABP4 in different subgroups.** (**A**) Serum levels of FABP4 in cardioembolic stroke and other stroke subtype groups. (**B**) Serum levels of FABP4 in obese and non-obese group. All data are medians and inter-quartile ranges (IQR). P values refer to Mann-Whitney U tests for differences between groups. FABP4= Fatty Acid Binding Protein 4.

A positive relationship was observed between serum FABP4 levels and stroke severity, since the stroke severity was evaluated with NIHSS score (r=0.493, P<0.001), Table 2. Furthermore, results indicated that FABP4 was correlated with lesion size, suggesting a positive relationship between FABP4 and the infarct volume (r=0.183, P=0.003). A modest correlation was also observed between FABP4 and Hs-CRP (r=0.141, P=0.043). However, no significant correlation was observed between FABP4 and gender, age and HCY (P>0.05 for all), Table 2. The correlation of FABP4 levels with stroke subtypes was also tested. The median FABP4 levels were higher for cardioembolic stroke [n=77] compared to those of other stroke subtypes [N=129] (18.9 [IQR, 14.0-27.8] vs. 17.3 [11.2-21.7] ng/ml; P=0.012; Figure 1A), with significance. In addition, FABP4 levels in obese patient (N=31) was higher than in non-obese (N=175) patients (21.9[IQR, 17.0-25.8] vs. 17.1[12.1-21.6]ng/ml; P<0.001), Figure 1B.

**Table 2:** The correlation between FABP4 and other factors.

Factors	r[spearman]	p
NIHSS	0.493	<0.001
Infarct volume	0.183	0.003
Hs-CRP	0.141	0.043
BMI	0.228	<0.001
Sex	0.086	0.366
age	0.127	0.092
HCY	0.093	0.269

Hs-CRP, High-sensitivity- C-reactive protein; NIHSS, National Institutes of Health Stroke Scale; HCY, homocysteine; FABP4; Fatty Acid Binding Protein 4.

**Figure 2:**
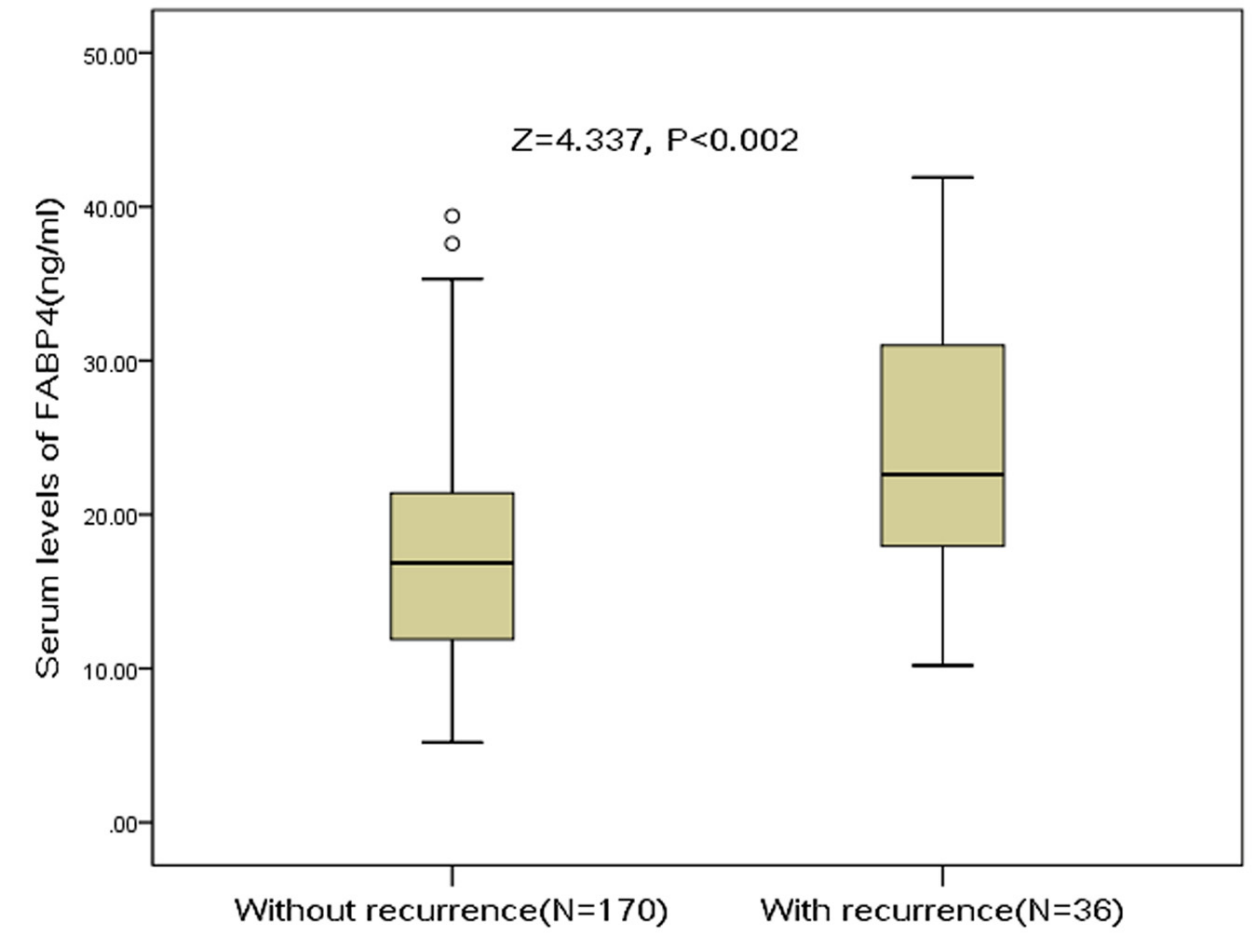
**Distribution of serum levels of FABP4 in ischemic stroke patients with stroke recurrence and without stroke recurrence.** All data are medians and inter-quartile ranges (IQR). P values refer to Mann-Whitney U tests for differences between groups. FABP4= Fatty Acid Binding Protein 4.

Stroke recurrence was observed in 36 patients (17.5%; 95%CI: 12.3%-22.7%). Median of serum FABP4 levels in patients with stroke recurrence was 22.6 (IQR, 17.9-31.6) ng/mL, which was higher than those of without stroke recurrence [16.9, (IQR, 11.8-21.4) ng/mL] (Figure 2). The difference was significant (P<0.001). The OR of serum FABP4 levels compared to NIHSS and other risk factors was calculated in logistic regression analysis. For each 1 ng/ml increment of FABP4 level in serum, the risk of stroke recurrence would be elevated by 12% in unadjusted model (with the OR of 1.12 [95% CI 1.06–1.17], P<0.001) and 8% in adjusted model (1.08 [1.02–1.14], P=0.006) (Table 2). In addition, NIHSS, stroke etiology, atrial fibrillation, HCY and Hs-CRP were also significant indicators (P<0.05 for all) (Table 3).

**Table 3:** Multivariate analysis of predictors of stroke recurrence ^‡^.

Predictors^†^	OR	95% CI	P
FABP4 BMI	1.08 1.19	1.02-1.14 0.98-1.66	0.006 0.36
Age	1.22	1.03-1.53	0.083
NIHSS	1.10	1.02-1.18	0.012
Infarct volume	1.07	0.84-1.36	0.59
Stroke etiology (Cardioembolic vs. other)	2.77	2.03-4.04	0.010
Acute treatment, TPA-T (yes vs. no)	0.58	0.47-0.67	0.002
Atrial fibrillation (yes vs. no)	2.06	1.07-3.91	0.029
HCY	1.07	1.00-1.13	0.038
Hs-CRP	1.21	1.02-1.43	0.027

^‡^Multivariable model included all of the following variables: age, sex, BMI, time from onset to blood collection, infarct volume, NIHSS score, stroke etiology, vascular risk factors, acute treatment, and serum levels of Hs-CRP**,** HCY and FABP4.

†Increased every one unit.

OR, odds ratio; CI, confidence interval; Hs-CRP, High-sensitivity- C-reactive protein; NIHSS, National Institutes of Health Stroke Scale; HCY, homocysteine; TPA-T: Tissue plasminogen activator-treated; FABP4; Fatty Acid Binding Protein 4.

**Figure 3:**
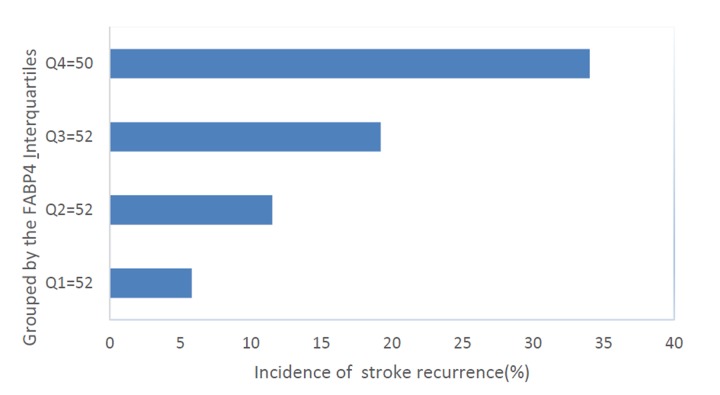
**The incidence for stroke recurrence in ischemic stroke according to the baseline FABP4 quartiles.** Serum levels of FABP4 in Quartile 1 (<12.7ng/ml), Quartile 2 (12.7–17.7ng/ml), Quartile 3 (17.8–22.8ng/ml), and Quartile 4 (>22.8ng/ml). FABP4= Fatty Acid Binding Protein 4.

The distribution of stroke recurrence across the FABP4 quartiles was ranged from 5.8% (Q1) to 34.0% (Q4) (Figure 3). For stroke recurrence, the OR of FABP4 Q4 against Q1 was 4.55 (95% CI, 2.03–12.15; P=0.001), while for Q2 and Q3, the OR was 1.77 (95% CI, 0.85–7.94; P=0.51) and 2.49 (95% CI, 0.95-10.12 P=0.12), respectively (Table 4). Furthermore, elevated serum levels of FABP4 (>22.8 ng/ml) were correlated with stroke recurrence, in which the risk was elevated by 272% (OR=3.72 [95% CI 1.74–7.92], P<0.001) and 129% (2.29 [1.31–5.11], P=0.003), respectively.

**Table 4:** Logistic regression model for serum levels of FABP4 quartiles using stroke recurrence as the dependent variables^‡^.

FABP4	SR/All, %	Unadjusted		Adjusted^‡^	
		OR (95%CI)	P	OR (95%CI)	P
Q1(<12.7ng/ml)	3/52, 5.8	Reference	—	Reference	—
Q2(12.7-17.7ng/ml)	6/52, 11.6	2.13(0.50-9.02)	0.30	1.77(0.85-7.94)	0.51
Q3(17.8-22.8ng/ml)	10/52, 19.2	3.89(1.00-15.07)	0.038	2.49(0.95-10.12)	0.12
Q4(>22.8ng/ml)	17/50, 34.0	8.41(2.28-31.01)	<0.001	4.55(2.03-12.15)	0.001
Elevated vs. normal^†^	17/50 vs. 36/170	3.72(1.74-7.92)	<0.001	2.29(1.31-5.11)	0.003

^‡^Adjustment by age, sex, BMI, time from onset to blood collection, infarct volume, NIHSS score, stroke etiology, vascular risk factors, acute treatment, and serum levels of Hs-CRP and HCY.

^†^ Elevated serum level of FABP4 was defined as >22.8ng/ml (3^rd^ quartile).

OR, odds ratio; CI, confidence interval; BMI, body mass index; NIHSS, National Institutes of Health Stroke Scale; Hs-CRP, high C-reactive protein; HCY, homocysteine; FABP4; Fatty Acid Binding Protein 4.

SR, stroke recurrence.

**Figure 4:**
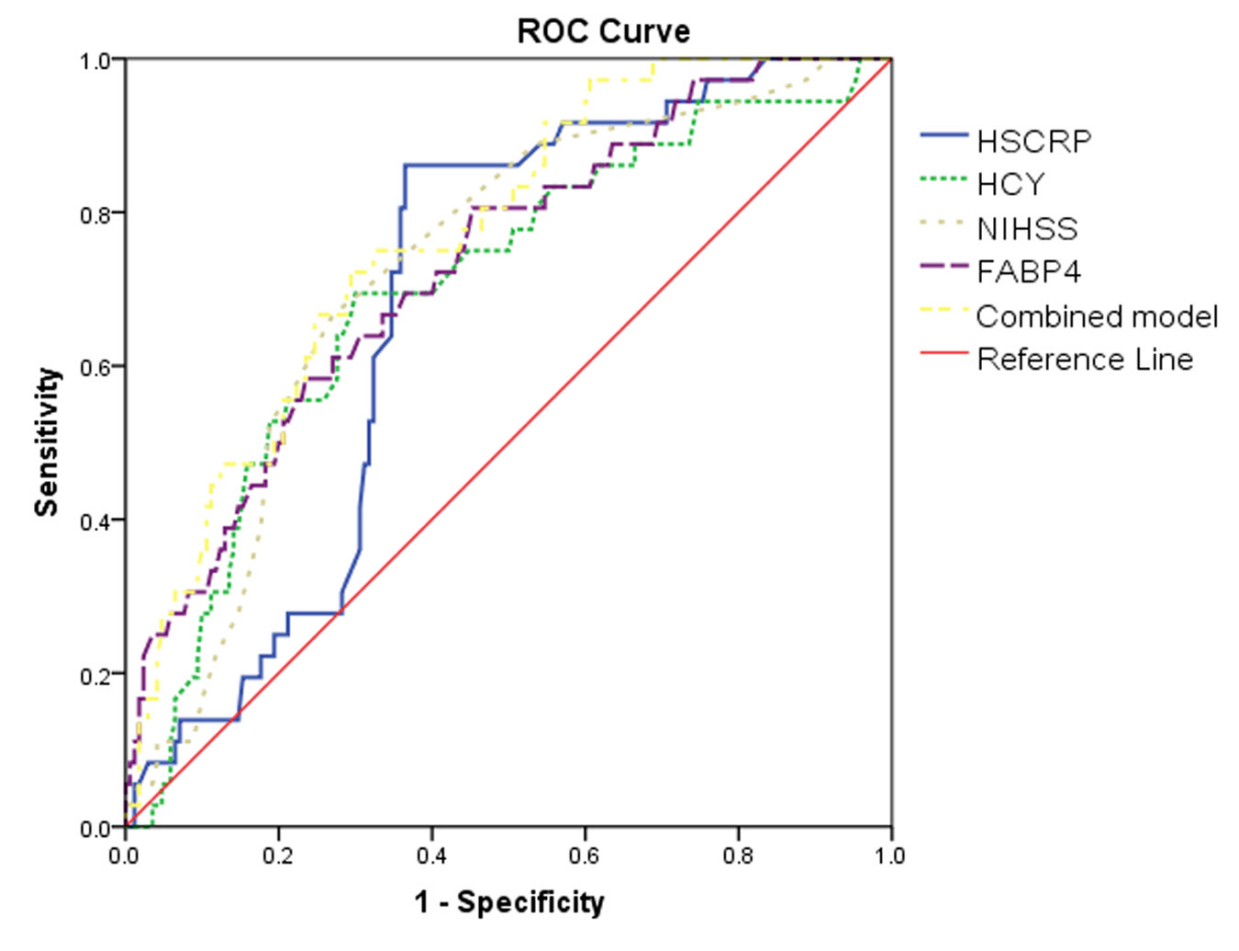
**Receiver operator characteristic curve demonstrating sensitivity as a function of 1 specificity for predicting the stroke recurrence.** Based on the logistic model incorporating 2 biomarkers (FABP4/NIHSS) and the relative contribution of each biomarker alone (FABP4/NIHSS/Hs-CRP/HCY). NIHSS=National Institutes of Health Stroke Scale; Hs-CRP=High C-reactive protein; HCY=Homocysteine; FABP4=Fatty Acid Binding Protein 4.

The discriminating capacity of FABP4 for predicting stroke recurrence was evaluated with AUC (0.73, 95% CI 0.64–0.82). The result was compared with Hs-CRP (AUC 0.69; 95% CI 0.61–0.77; P=0.02), HCY (AUC 0.70; 95% CI 0.61–0.80; P=0.03) and NIHSS score (AUC 0.72; 95% CI 0.64–0.81; P=0.45) (Table 5 and Figure 4). Interestingly, in a model combining NIHSS score and FABP4, the AUC was increased and reached 0.77 (95% CI, 0.69–0.85), indicating that NIHSS score could be significantly improved with FABP4 (P=0.01).

**Table 5:** Area under the curve for selected predictors of stroke recurrence.

Predictors	Stroke recurrence	
	ROC area	P
FABP4	0.73(0.64-0.82)	—
Hs-CRP	0.69(0.61-0.77)	0.02
HCY	0.70(0.61-0.80)	0.03
NHISS Score	0.72(0.64-0.81)	0.45
Model 1(FABP4+NIHSS)	0.77(0.69-0.85)	0.01

NIHSS, National Institutes of Health Stroke Scale; Hs-CRP, high C-reactive protein; HCY, homocysteine; FABP4; Fatty Acid Binding Protein 4; ROC, Receiver operating characteristic.

The FABP4-improved discriminating capacity was further confirmed with the internal 5-fold cross validation. The AUC (mean ± SD) was 0.73± 0.046 and 0.77± 0.040 for the NIHSS and the combining model, respectively, amount to a difference of 0.04± 0.006). The 5-fold cross-validated mean squared prediction error was 0.209 ± 0.014 and 0.196 ± 0.014 for the NIHSS and the combining model, respectively, amount to an average decrease of 0.013 ± 0.004). Further, higher discriminating capacity was observed in a model combining known risk factors and FABP4, compared to the model only involving known risk factors without FABP4 (P=0.009).

### Subgroup analysis

In the analysis, those patients with endpoint of all-cause death (N=35) were taken into account. A significantly higher discriminating capacity was still observed for FABP4 to predict stroke recurrence (AUC, 0.75; 95% CI, 0.70–0.81), compared to Hs-CRP (AUC 0.70; 95% CI, 0.62–0.76; P=0.009), HCY (AUC 0.71; 95% CI, 0.63–0.78; P=0.01) and NIHSS score (AUC 0.77; 95% CI, 0.70–0.83; P=0.09).

In addition, we also conducted analyses separately among patients who defined as obese (BMI≥30kg/m^2^) and non-obese. In the multivariate analysis, the date showed that for each 1 ng/ml increase of FABP4, the association between FABP4 and stroke recurrence was stronger among patients who defined as obese (OR= 1.14, 95%CI: 1.09-1.22; P<0.001) versus non-obese (OR=1.06, 95%CI: 1.02-1.14; P=0.016).

## DISCUSSION

FABP4, a third adipokine, functions as a critical mediator for inflammation in macrophages [22]. Peeters et al. [23] demonstrated the correlation between FABP4 levels and unstable plaque phenotype in atherosclerotic lesions, as well as the elevated risk of cardiovascular diseases in the follow-up. Our study has been the first study showed that nondiabetic stroke patients with elevated levels of FABP4 were more likely suffered from stroke recurrence in the future. The dose-dependent correlation was independent of existing stroke risk factors.

The associations between FABP4 and stroke had been proposed in recently studies. Tu et al. [24], showed that FABP4 could improve existing risk stratification for patients with stroke as an independent predictor. While another study performed in type 2 diabetes patients with acute ischemic stroke confirmed that increased level of FABP4 was related to an elevated risk of poor outcomes [25]. High FABP4 levels had been related to risk and severity in patient with stroke [26]. Furthermore, serum FABP 4 concentrations were closely correlated with peripheral arterial disease in Chinese women with T2DM [27], while another study reported the association between serum FABP 4 concentration and prognosis of patients with stable angina undergoing percutaneous coronary intervention [28]. In addition, serum A-FABP level was a biomarker of future poor cardiovascular outcomes in patients with coronary artery disease [22]. For the hypertensive patients, elevated FABP4 concentration was seen as a predictor for MetS and arterial stiffness [29]. These results revealed that FABP4 played multiple roles in stroke, which may be a promising target for treating these diseases.

The stroke recurrence was generally occurred within days and weeks after an ischemic stroke [30]. Previous community-based studies had suggested that the 3-year cumulative risk of stroke recurrence varied from 6 to 25% [31]. A previous study in Chinese stoke patients reported a recurrence rate of 10.6% in the 3-month follow-up [30]. Our study reported that 17.5% of stroke patients suffered from recurrent stroke, which was higher than in previous two studies (4.2% (95% CI, 3.2%-5.2%) [32] and 4.2% (2.8–6.2%) [33], respectively). Those differences might be resulted from study conditions, ethnicities, follow-up, diagnostic approach and disease types. The conditions of stroke recurrence are significant epidemiological data for evaluating our primary prevention, hospital care quality and secondary prevention [34].

Interestingly, a variety of studies have demonstrated that serum FABP4 levels are positively correlated with BMI [35,36]. Similarly, in this study, we also showed that FABP4 levels are positively correlated with BMI, and obese patients had significantly (P < 0.001) higher FABP4 concentrations compared with nonobese patients. However, in the multivariate analysis, serum FABP4 still was a positive risk factor for stroke recurrence independent from BMI in non-diabetic patients. Insulin resistance is linked to increased lipolysis [37], and secretion of FABP4 into the serum is stimulated by lipolysis [38]. Thus, stroke recurrence would be correlated with serum free fatty acid (FFA) levels. Interestingly, Choi et al. [39] confirmed this hypothesis, and suggested that an elevated FFA concentration could be a useful indicator for predicting recurrent stroke in cardioembolic stroke patients. Therefore, the association between FFA and stroke recurrence in non-diabetic stroke patients need to be further explored.

The mechanism of the positive association between FABP4 level and stroke recurrence risk was uncertain. Frist, recent study indicated that FABP4 was related to the increased cardiometabolic risk. Previous studies investigated the correlation between FABP4 and atherosclerosis, as well as coronary artery disease. FABP4 was directly associated with cardiac diseases, including left ventricular hypertrophy, systolic and diastolic cardiac dysfunction [40]. Increased FABP4 levels in serum was also significant correlated with a greater coronary plaque burden [41]. Second, FABP4 lead to increased blood pressure in patient with hypertension, as well as atherogenic metabolic phenotype [42], another study found that increased second trimester plasma FABP4 independently predicted gestational hypertension or preeclampsia in gestational diabetes mellitus patients [43]. Third, FABP4 was derived from fat cells, making effects on regulating the transport of non-esterified fatty acid and peroxisome proliferator-activated receptor γ agonists. Meanwhile, both the lipid metabolism and insulin sensitivity would be influenced by interaction of FABP4 and proteins [22]. Fourth, FABP 4 may affect the endothelial cells proliferation and further angiogenesis. The aP2 played a critical role in the regulation of lipid-induced macrophage endoplasmic reticulum (ER) stress. The production of FABP4 in macrophage can be prevented with a chemical chaperone for reducing ER stress [44]. Lastly, FABP4 induced proinflammatory and proatherogenic cytokines in macrophages. One study reported that LPS-induced inflammatory responses could be enhanced with FABP4, by forming a finely-tuned positive loop between c-Jun NH2-terminal kinases and activator protein-1 [45]. FABP4 was reported to make important effects on the inflammation in metabolic alterations [46]. In addition, FABP4 was known to function in inflammation by virtue of its ability to regulate intracellular events such as lipid fluxes and signaling [47]. After suppressing FABP4 signaling, a protective effect was observed in mouse model with an acute lung injury (ALI). The lipopolysaccharide induced FABP4 expression in A549 cell would lead to increased level of reactive oxygen species, thus causing inflammatory cytokine production [48]. Interestingly, some inflammation biomarkers, such as macrophage Migration Inhibitory Factor [49] and lipoprotein (a) [50] had been suggested to be involved in stroke recurrence.

Previous studies had showed that circulating levels of FABP4 were associated with stroke risk, severity [26] and functional outcome [24] in patients with acute ischemic stroke. The prognostic value of FABP4 in in patients with type 2 diabetes and acute ischemic stroke also had been suggested [25]. However, associations between serum FABP4 levels and stroke recurrence had not been explored. In addition, in this study, patients with diabetes had been excluded. We could assess the real relationship between the FABP4 and stroke recurrence to exclude the effects caused by metabolic abnormalities. In addition, a variety of statistical methods had been used to assess the prognostic value of FABP4.

Furthermore, some limitations should be presented. First, the project design of small sample (N=206) and single center cannot produce effective results. In addition, there were only 36 recurrent strokes, leading to a relatively limited power. Thus, the adjustment in logistic regression may be too extensive with overfitting. Second, circulating FABP4 levels were determined with a single measurement at baseline. Without serial testing of FABP4 levels, we yielded no data regarding the change of FABP4 levels in those nondiabetic stroke patients. Interestingly, one study reported significantly elevated FABP4 levels during the early hours after the occurrence of acute myocardial infarction [51]. In addition, cerebrospinal fluid samples were not obtained. Third, there were no data for other adipokine, such as leptin, omentin and retinol binding protein 4 (RBP4). The association between FABP4 and those factors should be clarified. Hence, the true association between FABP4 and stroke recurrence might be caused by those adipokines confounds. Fourth, some diabetes markers, such as Homeostatic Model Assessment of Insulin Resistance (HOMA-IR) and Hemoglobin A1c (HbA1c) might affect the predictive effectiveness of FABP4 in the non-diabetic patients. However, in this study, we did not test those factors due to the diabetic patients had been excluded. Thus, the impact of those factors on stroke recurrence in relation to the serum FABP4 remains unclear. Interestingly, Tu et al. [24] reported that serum FABP4 was a positive risk factor for stroke outcome independent from HOMA-IR and HbA1c. Lastly, any causal relationship could not be suggested due to the cross-sectional study design. What’s more, the patients with the secondary endpoint of all-cause death were not included in the analysis, who may also experience stroke recurrence. Therefore, the results can be under-estimated. The association between FABP4 and mortality was not evaluated, since the mortality data was not collected in current study.

In summary, this cohort study was performed on nondiabetic patients with ischemic stroke. The results indicated that increased FABP4 level in serum was related to higher risk of early stroke recurrence in the future, independent of baseline variables. Because it makes critical effects at multiple stages of stroke development, FABP4 will be worthy of further research as a possible therapeutic target.

## PATIENTS AND METHODS

### Patients and examination

During December 2016 and May 2018, this study included the consecutive patients hospitalized in Beijing Tian Tan Hospital, Capital Medical University (China) due to the acute ischemic stroke. Acute ischemic stroke was determined per the recommendations of World Health Organization recommendations (cerebrovascular diseases caused neurological deficit, which lasted for more than 24 hours or died within 24 hours) [52]. The diagnose was confirmed with MRI within 24 hours after the hospitalization. The exclusion criteria were as follows: (1) malignancy; (2) diabetes mellitus (the medical record reported glucose level > 7.8 mM or patient under the treatment of insulin or oral hypoglycemic agents) and/or metabolic syndrome; (3) symptoms onset more than 24h at admission; (4) liver and kidney function dysfunction; (5) chronic coexistent inflammatory disease or other neurologic diseases.

### Clinical variables

The information was collected, including age, gender, body mass index (BMI; obese was defined as BMI≥30kg/m^2^), vascular risk factors (Table 1 for details). Acute treatment was also and recorded, such as Intravenous [IV] thrombolysis and/or mechanical thrombectomy. The score of National Institute of Health Stroke Scale (NIHSS) [53] was evaluated and conducted by a certified stroke neurologist (Li HM). The stroke etiology was identified with Trial of Org 10172 in Acute Stroke Treatment (TOAST) criteria according to previous study [54]. MRI was performed for partial patients. The infarct volume was obtained according to the MRI image, with the formula of *0.5 × a × b × c*, where *a* was the maximal longitudinal diameter, *b* was the maximal transverse diameter perpendicular to *a*, and *c* was the number of 10-mm slices containing infarct [55].

### Follow-up and stroke recurrence

For all the patients, the follow-up was performed with a standard questionnaire, telephone or visit (Jiang P). Median follow-up was 3 months. Stroke recurrence was the primary endpoint, defined as suddenly deteriorated neurological function evaluated as a decreased NIHSS score of 4 or more, or a new focal neurological deficit of vascular origin lasted for >24 hours [56]. All-cause death was the secondary endpoint, defined as death due to any causes. The patients end up with the all-cause death in the follow-up were excluded [30].

### Laboratory testing

Fasting blood of patients were drawn at 8:00 on the first hospitalization day for testing levels of FABP4. The serum FABP4 was detected with a commercial ELISA kit (R&D Systems, Minneapolis, MN). For this assay, the determination range was 1.5-100 ng/ml. The coefficient of variation intra- and inter-assay was less than 10.0% in the range of 100-10 ng/ml. In addition, other serum biomarkers were also detected, such as C-reactive protein (Hs-CRP) and homocysteine (HCY).

### Statistical analyses

The results were expressed as percentages for categorical variables and as medians (interquartile ranges [IQRs]) for continuous variables. The comparison was conducted with Mann-Whitney U test for continuous variables and with chi-square test for categorical variables. Bivariate correlation was assessed by the Spearman's rank correlation.

The association between FABP4 and stroke recurrence was analyzed with logistic regression models. The odds ratios (ORs, 95% confidence intervals [CI]) were analyzed with both unadjusted and adjusted model. In addition, the association between stroke recurrence and FABP4 quartiles was explored, with the 1^st^ quartile as reference. The multivariate analysis model was applied for further estimating the adjusted OR (95%CI). Further, the relationship between high FABP4 (defined as >3^rd^ quartile=22.8ng/ml) and stroke recurrence was also presented.

Second, the risk of stroke recurrence obtained with various models were compared. The receiver operating characteristic curve (ROC) was plotted and the area under the curve (AUC) was calculated for obtaining criteria and cut-point. To test if the efficiency could be improved with FABP4 level, two nested logistic regression models were applied. In addition, optimistic bias of in-sample prediction error estimates was carefully adjusted with a fivefold cross-validation scheme [57].

The statistics were analyzed with SPSS, version 22.0. The ROC was plotted with ROCR package, version 1.0-2 (CRAN repository, http://cran.r-project.org/). P<0.05 was considered to be significant.

### Ethics

This study has been reviewed and approved by the ethics committee of the Beijing Tian Tan Hospital, Capital Medical University. The study protocol was introduced and interpreted to all the patients and informed consents of all the involved patients were obtained.

### Ethics, consent and permissions

Written informed consents were obtained from all patients; and, this study conformed to the principles of the Declaration of Helsinki was approved by the investigational review board of the Beijing Tian Tan Hospital, Capital Medical University.

### Data Availability

Please contact author for data requests.
